# DEB025 (Alisporivir) Inhibits Hepatitis C Virus Replication by Preventing a Cyclophilin A Induced Cis-Trans Isomerisation in Domain II of NS5A

**DOI:** 10.1371/journal.pone.0013687

**Published:** 2010-10-27

**Authors:** Lotte Coelmont, Xavier Hanoulle, Udayan Chatterji, Carola Berger, Joke Snoeck, Michael Bobardt, Precious Lim, Inge Vliegen, Jan Paeshuyse, Grégoire Vuagniaux, Anne-Mieke Vandamme, Ralf Bartenschlager, Philippe Gallay, Guy Lippens, Johan Neyts

**Affiliations:** 1 Department of Microbiology and Immunology, Rega Institute for Medical Research, University of Leuven, Leuven, Belgium; 2 Centre National de Recherche Scientifique – Unité Mixte de Recherche 8576, Université de Lille 1, Villeneuve d'Ascq, France; 3 Department of Immunology and Microbial Science, The Scripps Research Institute, La Jolla, California, United States of America; 4 Department of Molecular Virology, University of Heidelberg, Heidelberg, Germany; 5 Pharmacology and Screening Unit, Debiopharm, Lausanne, Switzerland; Yonsei University, Republic of Korea

## Abstract

DEB025/Debio 025 (Alisporivir) is a cyclophilin (Cyp)-binding molecule with potent anti-hepatitis C virus (HCV) activity both *in vitro* and *in vivo*. It is currently being evaluated in phase II clinical trials. DEB025 binds to CypA, a peptidyl-prolyl *cis-trans* isomerase which is a crucial cofactor for HCV replication. Here we report that it was very difficult to select resistant replicons (genotype 1b) to DEB025, requiring an average of 20 weeks (four independent experiments), compared to the typically <2 weeks with protease or polymerase inhibitors. This indicates a high genetic barrier to resistance for DEB025. Mutation D320E in NS5A was the only mutation consistently selected in the replicon genome. This mutation alone conferred a low-level (3.9-fold) resistance. Replacing the NS5A gene (but not the NS5B gene) from the wild type (WT) genome with the corresponding sequence from the DEB025^res^ replicon resulted in transfer of resistance. Cross-resistance with cyclosporine A (CsA) was observed, whereas NS3 protease and NS5B polymerase inhibitors retained WT-activity against DEB025^res^ replicons. Unlike WT, DEB025^res^ replicon replicated efficiently in CypA knock down cells. However, DEB025 disrupted the interaction between CypA and NS5A regardless of whether the NS5A protein was derived from WT or DEB025^res^ replicon. NMR titration experiments with peptides derived from the WT or the DEB025^res^ domain II of NS5A corroborated this observation in a quantitative manner. Interestingly, comparative NMR studies on two 20-mer NS5A peptides that contain D320 or E320 revealed a shift in population between the major and minor conformers. These data suggest that D320E conferred low-level resistance to DEB025 probably by reducing the need for CypA-dependent isomerisation of NS5A. Prolonged DEB025 treatment and multiple genotypic changes may be necessary to generate significant resistance to DEB025, underlying the high barrier to resistance.

## Introduction

Worldwide more than 170 million people are chronically infected with HCV and at increased risk to develop liver cirrhosis and/or hepatocellular carcinoma [1]. The current standard of care consists of a combination of pegylated interferon alpha (pegIFN-α) and ribavirin (RBV), administered for a period of 24 to 48 weeks depending on HCV genotype. This therapy is however associated with serious side effects and sustained virological response rates are unsatisfactory, particularly for HCV genotype 1 infection [2]. Direct acting antivirals (DAA), i.e. molecules that target for example the HCV NS3 protease and NS5B polymerase have been discovered and several are in clinical development [3]. Alternatively, host cell factors that are essential for efficient HCV replication can be targeted. The immunosuppressive drug CsA has been reported to exert anti-HCV activity *in vitro* and *in vivo* [4]–[6]. The anti-HCV activity of CsA has been shown to be linked to its ability to interact with cyclophilins (Cyps), which were identified as crucial cellular cofactors for HCV replication. Cyps display a peptidyl-prolyl *cis-trans* isomerase activity (PPIase) that catalyzes the *cis-trans* isomerisation of the prolyl peptide bond preceding proline residues. Data concerning the Cyp subtype essential for HCV replication are controversial. CypB has been suggested to act as a functional regulator of the HCV RNA polymerase by enhancing its RNA-binding affinity and subsequently increasing the RNA polymerase activity [7]. Others reported that CypA, B and C are indispensible for HCV replication [8]. More recently there is a growing consensus that in particular CypA is a crucial factor during HCV replication [9]–[11]. A number of point mutations in NS5B and NS5A have been reported to be associated with *in vitro* resistance to CsA [12]–[15]. Direct interactions between CypA and NS5B or NS5A have been observed [11], [16]–[18].

Several CsA-analogues, i.e. NIM811 [19], DEB025 and SCY-635 [20], are currently in preclinical and clinical development. These molecules retain the binding affinity for Cyp, but do not inhibit calcineurin, which is the molecular target underlying the immunosuppressive activity of CsA [21]. We reported earlier on the potent *in vitro* anti-HCV activity of DEB025 [22] and on the particular characteristics of its anti-HCV activity [23]. During phase I clinical studies in HCV/HIV-coinfected patients, DEB025 monotherapy (1200 mg BID) resulted in a mean maximal decrease in HCV viral load of −3.6 log_10_ IU/ml after 15 days of therapy [24]. The efficacy and safety of DEB025 was further evaluated in a phase II study in which HCV genotype 1, 2, 3 and 4 treatment–naïve patients were randomized to receive escalating doses of DEB025 (200, 600, 1000 mg/day) combined with pegIFN-α-2a or monotherapy of either drugs. In patients with genotype 1 and 4, therapy based on the 1000 mg/day dose of DEB025 resulted in a decrease in viral load of −4.75 log_10_ IU/ml. Viral load reduction in genotype 2 and 3 patients was even more pronounced with a decrease up to −5.89 log_10_ IU/ml [25]. Whereas lower doses regimens are very well tolerated, the 1000 mg dose was associated with isolated and transient hyperbilirubinemia which returned to baseline level after treatment cessation. Subsequently, DEB025 was investigated in combination with pegIFN-α-2a and RBV in HCV genotype 1 null/partial responders to pegIFN/RBV for 29 days. DEB025, at doses of 400 mg (with initial loading dose) and 800 mg daily, resulted, when combined with pegIFN/RBV, in a viral load reduction of −1.96 to −2.38 log_10_ IU/ml, respectively [26].

We here report on the *in vitro* selection and characterization of HCV subgenomic replicons (GT1b) resistant to DEB025. We propose a mechanism by which the cyclophilin inhibitors prevent HCV replication and how resistant replicons may interfere with this inhibition.

## Results

### *In vitro* resistance selection and cross-resistance profiles of DEB025 and CsA

**Figure 1:**
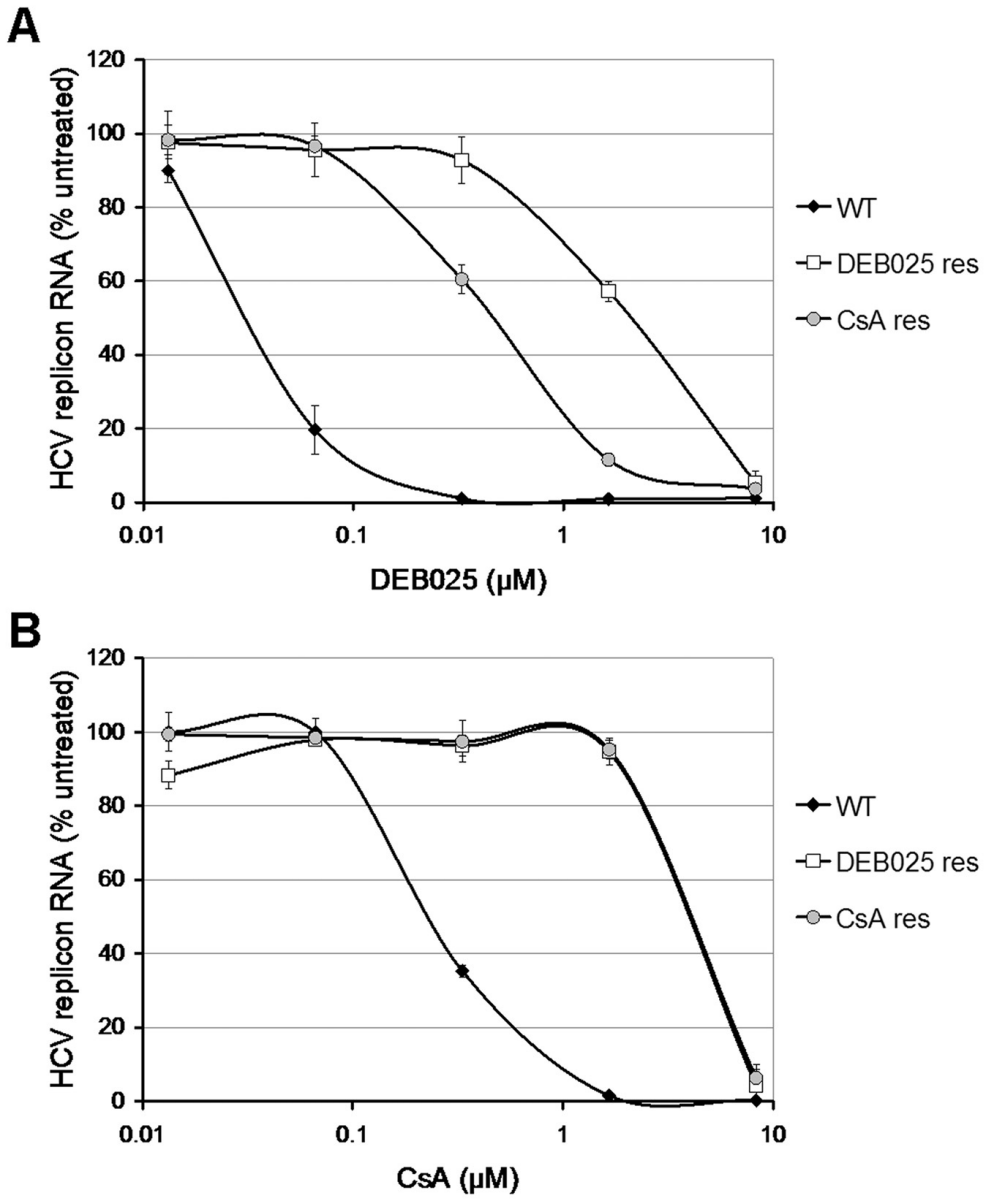
Characterization of DEB025^res^ or CsA^res^ replicon containing cell lines. Dose-response curves for inhibition of viral subgenomic replication by either (A) DEB025 or (B) CsA in WT (closed diamonds), DEB025^res^ (open squares) and CsA^res^ (grey circles) cell lines. The respective Huh 9-13 cells were treated for 72 h with escalating concentrations of either DEB025 or CsA. HCV replicon RNA was quantified by means of RT-qPCR and is expressed as the percentage of HCV replicon RNA of the untreated control cells. Data are mean values±standard deviations for at least three independent experiments.

Huh 9–13 cells (Con1-GT1b) were cultured (in G418-containing medium) in the presence of an initial concentration of 0.21 µM of DEB025 or 0.42 µM of CsA. Higher concentrations of DEB025 resulted in rapid elimination of the HCV replicon from the cells. Replicon-containing cells were continuously passaged in these double-selection media. When cultures had adapted to replication in the presence of a particular concentration of DEB025 (i.e. no more massive cell death) the concentration was increased in steps of 0.21 µM (typically every 3 passages). Finally, replicon-containing cells were obtained that replicated in the presence of 2.05 µM DEB025 (approximately 65-fold the EC_50_-value of DEB025), a concentration that completely inhibits WT replicon replication, or 2.5 µM CsA (approximately 10-fold the EC_50_-value of CsA). As on average 35 passages (∼20 weeks) and 40 passages (∼23 weeks) of selective pressure were needed, for DEB025 and CsA respectively, the barrier to resistance appears to be high for cyclophilin inhibitors. DEB025 and CsA proved cross-resistant, as the DEB025^res^ culture was markedly less sensitive to both DEB025 (EC_50_: 2.05±0.56 µM) and CsA (EC_50_: 3.67±0.78 µM) as compared to WT (EC_50_: 0.03±0.01 µM and 0.23±0.03 µM, respectively) (Figure 1). Likewise the CsA^res^ culture proved markedly less sensitive to both DEB025 and CsA (EC_50_: 0.46±0.26 µM for DEB025 and 3.77±0.63 µM for CsA). However, both of them retained WT-activity against replicons resistant to DAA inhibitors of different classes [23]. Conversely, HCV polymerase inhibitors (2′-*C*-methylcytidine and 4′-azidocytidine) and protease inhibitors (BILN2061 and VX-950) retained WT-antiviral activity against DEB025^res^ and CsA^res^ replicons [23], indicating a lack of cross-resistance between DEB025 and DAAs of other classes.

### *In vitro* resistance to DEB025 is mediated via the viral genome

**Figure 2:**
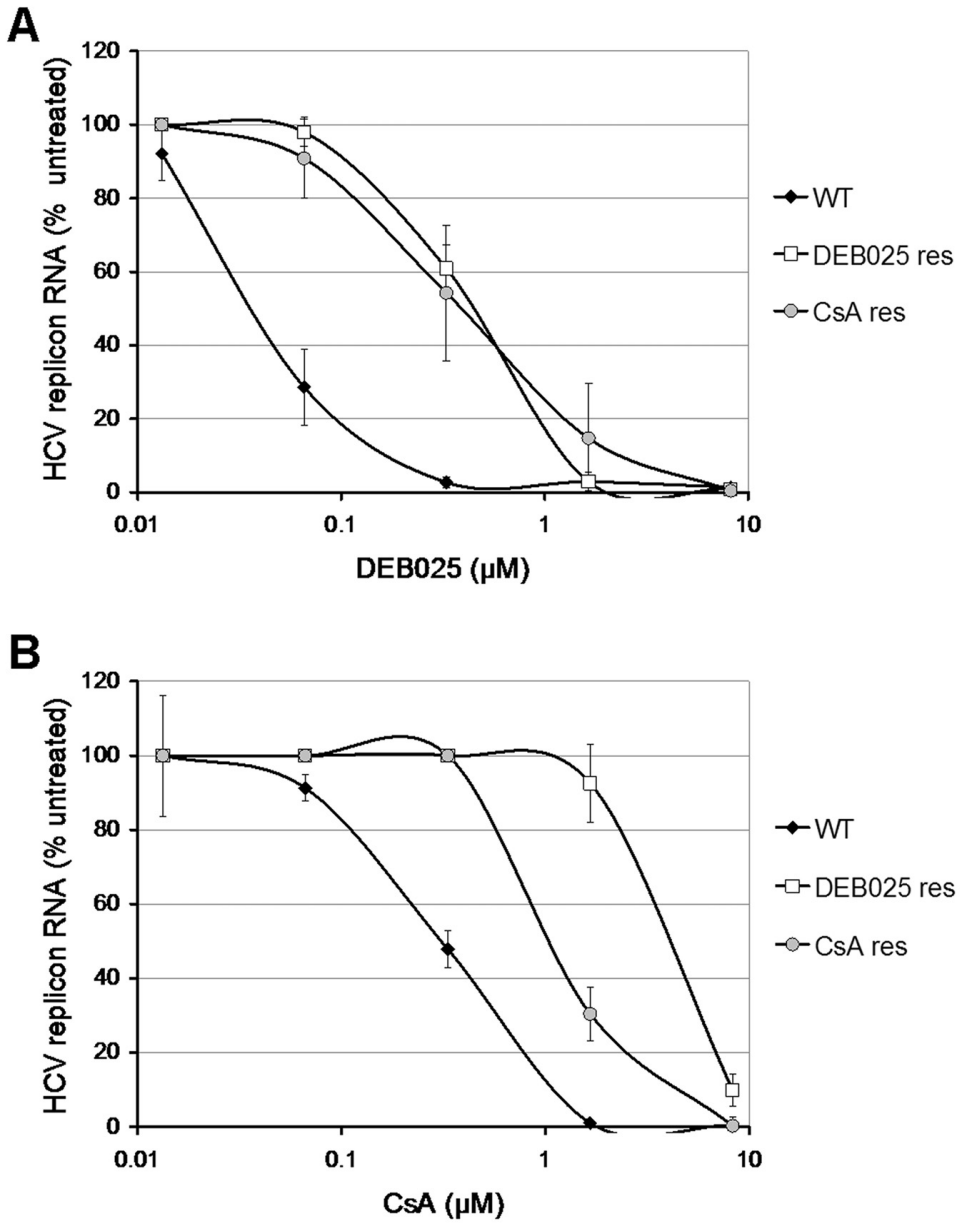
Drug resistance is associated with the viral genome. Dose-response curves for inhibition of replicon RNA by either (A) DEB025 or (B) CsA in Huh7-Lunet cells stably transfected with RNA isolated from WT (closed diamonds), DEB025^res^ (open squares) and or CsA^res^ (grey circles) Huh 9–13 cell lines. HCV replicon RNA was quantified by means of RT-qPCR and is expressed as the percentage of HCV replicon RNA of the untreated control cells. Data are mean values ± standard deviations for at least three independent experiments.

To study whether the factor that determines drug resistance was associated with the viral genome or with the host cell, RNA isolated from DEB025^res^, CsA^res^ and WT cultures (the latter cultured in parallel with the resistance selection) was transfected into naïve Huh7-Lunet cells by means of lipofection. Following three weeks of G418 selective pressure stable cell lines were obtained and subsequently phenotyped. DEB025 proved on average 10-fold less active in cells stably transfected with RNA isolated from the DEB025^res^ culture (EC_50_: 0.44±0.07 µM), as compared to the cell line transfected with WT replicon (EC_50_: 0.04±0.01 µM) (Figure 2A). CsA proved on average 3.5-fold less active in the cell line obtained following transfection with RNA isolated from the CsA^res^ culture, as compared to WT (EC_50_: 1.08±0.12 µM and 0.31±0.05 µM, respectively) (Figure 2B). Furthermore, cross-resistance between DEB025 and CsA was confirmed. Of note, when stable cell lines were generated in the absence of DEB025 or CsA, the mutant replicons retained their original mutations and did not revert back to the WT sequence (data not shown). Since the cell lines transfected with RNA from DEB025^res^ and CsA^res^ cultures proved also resistant to the drugs, although not to the full extent as the originally selected resistant cultures; it is concluded that resistance is at least in part associated with the viral genome, although cellular changes may also play a role as discussed below.

The P-glycoprotein [P-gp, the product of the multidrug resistance (MDR) gene, is an ATP-dependent pump that extrudes certain drugs from the cell] content of these cell lines was determined by means of FACS analysis. P-gp was on average 9-fold more abundant on the DEB025^res^ cells and 7.5-fold more on the CsA^res^ cells, as compared to WT cells (data not shown). Whether these cellular changes play any role in resistance to DEB025 and CsA remains to be determined. We will focus our attention in this manuscript on the resistance associated with the viral genome.

### NS5A D320E confers low-level resistance to DEB025

**Figure 3:**
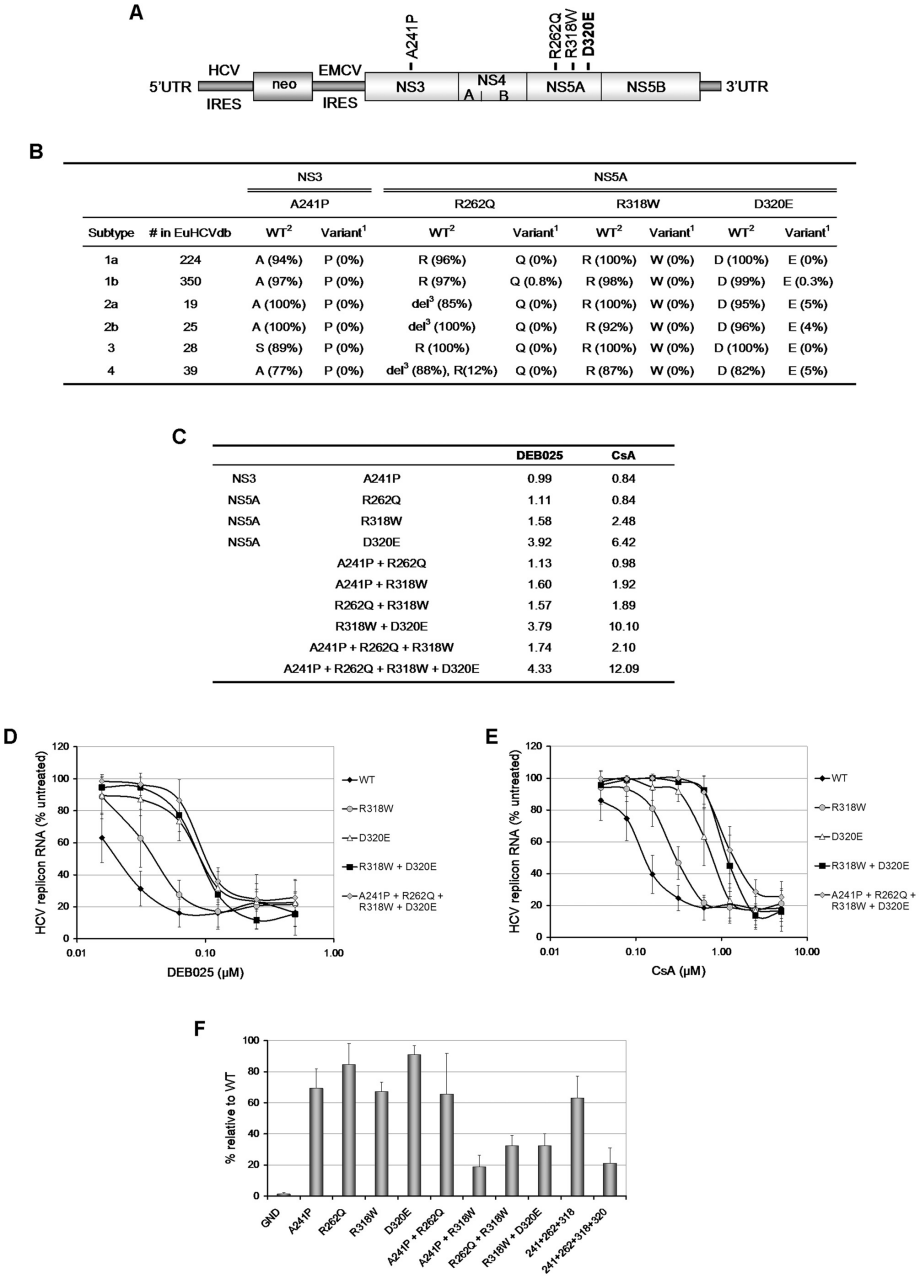
Identification and characterization of mutations conferring resistance to DEB025. (A) Schematic representation of the selectable Con1 (GT1b) subgenomic replicon (Huh 9–13) that was used to select for DEB025 (and CsA) resistance. RNA isolated from drug resistant and control cell cultures (passaged in parallel), was sequenced. The mutations identified in the resistant cultures are depicted above their respective position in the polyprotein. (B) Prevalence of the variants, found in the replicon after DEB025 selection, in different genotypes in the European HCV database. ^1^Variant identified in the DEB025^res^ replicon. ^2^WT amino acid for that genotype (the amino acid with the highest prevalence). ^3^del = deletion, [deletion, all of the genotype 2b and most of the genotype 2a and 4 sequences miss amino acid 262 (numbering according to genotype 1b) in NS5A]. (C) Mutations, that had been identified in the DEB025^res^ or CsA^res^ replicon, were introduced (either alone or combined) in a WT background, after which the sensitivity to DEB025 and CsA was assessed. Data are expressed as fold reduction (based on EC_50_-values) in sensitivity to the drug as compared to WT replicon. (D) Dose-response curves for inhibition of replicon replication by DEB025 or (E) CsA in Huh7-Lunet cells transiently transfected with mutant replicon RNA (indicated on the right site of each panel). HCV replicon RNA was quantified by means of a luciferase assay and data are expressed as percentage of untreated controls. Data are mean values ± standard deviations for at least three independent experiments. (F) Replication fitness of the various mutant replicons. Huh7-Lunet cells were transiently transfected with the indicated mutant replicons and RNA replication was measured by means of a luciferase assay for 4 days post transfection. Data are normalized to the 4 h-value post electroporation to account for differences in electroporation efficiency and expressed as a percentage of the WT-value at 96 h post electroporation. Data are mean values ± standard deviations for at least two independent experiments.

To identify the variations in the viral genome associated with the observed resistant phenotype, we sequenced the entire subgenomic region encoding the non-structural proteins of the replicon RNA from DEB025^res^ and CsA^res^ cultures and compared them to the WT sequence of non-resistant control cells. Four mutations were identified (both by population as well as clonal sequencing) with one residing in the NS3 serine protease gene (A241P) and three in domain II of the NS5A gene (R262Q, R318W and D320E) (Figure 3A).

We determined the prevalence of these mutations in HCV genotypes (GT) 1a, 1b, 2a, 2b, 3 and 4 in the European HCV database (euHCVdb) [27] (Figure 3B) and Los Alamos HCV database (Table S2). The A241P variant in NS3 is observed in none of the sequences in the database. All genotypes have an A at this position, except for genotype 3, which has an S as wild type amino acid. In the NS5A gene, Q262 and W318 are equally absent in all sequences. All genotypes have the R at position 318. For position 262 genotype 1 and 3 have the R (as the replicon), while most of the genotype 2 and 4 sequences lack this position (based on an alignment with all genotypes). The D320E variant is present in a small number of sequences of genotype 1b, 2a, 2b and 4; all genotypes bear the D as wild type amino acid.

To study the contribution of the identified mutations to the resistant phenotype, single mutations and combinations thereof were reintroduced into a WT GT1b subgenomic luciferase replicon backbone via site-directed mutagenesis and the susceptibility of these constructs to DEB025 and CsA was determined (Figure 3C). Introduction of A241P or R262Q had no effect on the susceptibility to DEB025 or CsA. Introduction of R318W resulted in a ±2-fold change in sensitivity to DEB025 and CsA. D320E resulted in a more pronounced effect on the antiviral phenotype (3.92-fold for DEB025 and 6.42-fold for CsA). A genome carrying in addition to D320E also R318W or [R318W+A241P + R262Q] did not result in an additional reduction in sensitivity to DEB025 (3.79 and 4.33-fold WT, respectively) (Figure 3D). The D320E mutation, when combined with R318W, either with or without [A241P + R262Q], resulted in a phenotype that was about 10-fold less sensitive to CsA (Figure 3E). Again, cross-resistance between DEB025 and CsA was confirmed. The generated mutant replicons retained WT-activity to IFN, protease (VX-950) and polymerase (2′-*C*-methylcytidine and HCV796) inhibitors (data not shown). To exclude that the observed resistance is the result of generally enhanced replication, the replication fitness of each mutant was compared to that of WT replicon. The replicative capacity of the single mutated replicons was slightly reduced or comparable to that of WT, whereas replicons that carried two or more mutations had a reduced replicative fitness compared to WT (Figure 3F).

**Figure 4:**
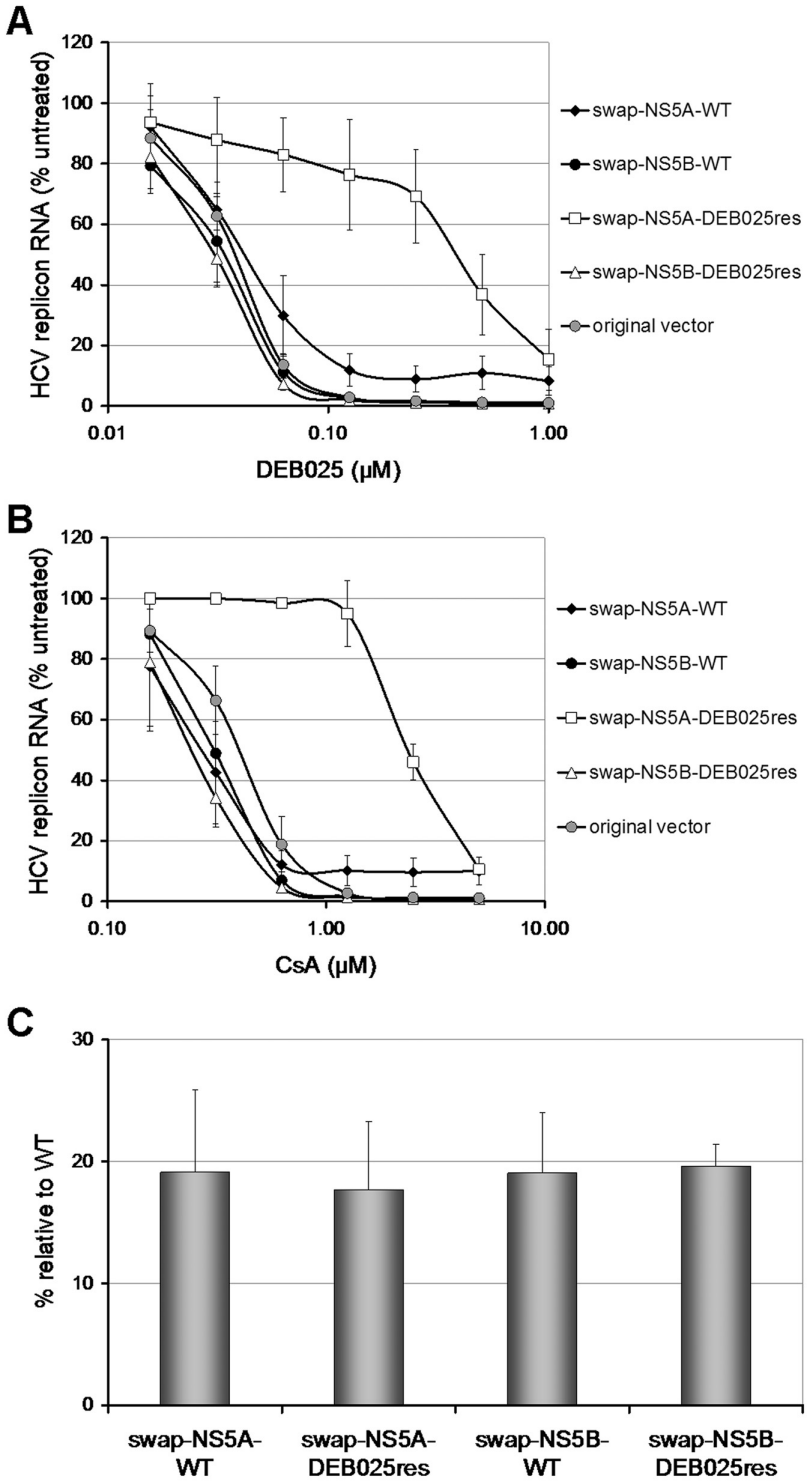
Mutations in NS5A but not NS5B contribute to resistance against DEB025. Dose-response curves for inhibition of replicon replication by (A) DEB025 or (B) CsA in Huh7-Lunet cells transiently transfected with recombinant replicon RNA carrying either the NS5A or NS5B region from DEB025^res^ or control (WT) cultures. For comparative reasons also the original unmanipulated vector was included in the analysis. Cells were treated for 72 h with escalating concentrations of DEB025 or CsA and HCV replicon RNA was quantified by means of a luciferase assay and is expressed as percentage of the untreated controls. Data are mean values ± standard deviations for at least three independent experiments. (C) Replication fitness of the different recombinant replicons. Huh7-Lunet cells were transiently transfected with each of the indicated replicon constructs and replicon RNA replication was quantified by means of a luciferase assay for 4 days post transfection. Data are normalized to the 4 h-value post electroporation to account for differences in electroporation efficiency and is expressed as percentage of the WT-value at 96 h post electroporation. Data are mean values ± standard deviations for at least two independent experiments.

The important role of the change from aspartic acid to glutamic acid at position 320 (D320E) in domain II of NS5A was further substantiated by the observation that this was the only common mutation found in three additional independent resistance selection assays performed in Huh 9–13 cells (two selections) and in Huh 5–2 cells (one selection) (Table S3). The majority of identified mutations clustered in the NS5A region. We therefore replaced the entire NS5A gene from the WT genome with the corresponding NS5A from the DEB025^res^ (1^st^ res selection) genome. The antiviral phenotype of this construct was evaluated in comparison with a construct into which the NS5A region derived from WT Huh 9–13 cells (which were cultured in parallel), was swapped. DEB025 proved 10-fold and CsA 9-fold less active in replicons that were engineered to contain the DEB025^res^ NS5A sequence compared to replicons that were engineered to contain the WT NS5A sequence (EC_50_: 0.39±0.15 µM *versus* 0.04±0.01 µM for DEB025 and 2.36±0.20 µM *versus* 0.28±0.08 µM for CsA) (Figure 4A and B).

It has been reported that CsA may select for I432V in NS5B and that NS5B might be involved in the mechanism of action of CsA [28]. In our hands, I432V neither reduced the antiviral activity of DEB025 or CsA nor enhanced the resistance observed with the D320E mutation (Figure S1). We also introduced the entire NS5B gene of the DEB025^res^ replicon in a WT background, as well, by means of control, the NS5B region from WT Huh 9–13 cells (which were cultured in parallel). No reduction in antiviral activity of DEB025 and CsA was observed (Figure 4A and B). The NS5A and NS5B swapped constructs retained WT-sensitivity to several protease and polymerase inhibitors (data not shown). Taken together the swapping results corroborate the observation that mutations in NS5A are responsible for the observed genome-associated resistance. The replication fitness of all swapped constructs was reduced compared to that of the ‘unmanipulated’ original vector (Figure 4C).

Recently we showed that JFH1-derived subgenomic replicon (GT2a) with a ∼5-fold reduced sensitivity to DEB025 carried a mutation near the cleavage site (position 464) between NS5A and NS5B. This mutation was demonstrated to slow down cleavage kinetics, implying a correlation between HCV polyprotein processing and CypA dependence of HCV replication [10]. Introduction of these mutations (V445A and V445L are the corresponding mutations in the GT1b background) into the GT1b subgenomic replicon resulted in a 1.28-fold change in sensitivity to DEB025 for V445A and 1.93-fold for V445L compared to WT (two independent experiments; Figure S2).

### DEB025^res^ replicon is less dependent on CypA for efficient replication

**Figure 5:**
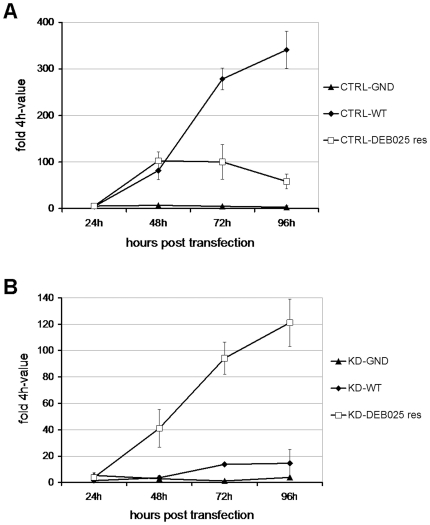
RNA replication of DEB025 resistant replicon is less dependent on CypA. Replication competence of replicon RNA isolated from DEB025^res^ or WT Huh 9–13 cultures in Huh7-Lunet cells (A) transduced with a lentiviral control vector (CTRL) or with (B) a stable knock down of CypA (KD). Replication was quantified at various time points post electroporation by means of RT-qPCR. A replication-deficient construct (GND) was included as a negative control. Data are expressed relative to the 4 h-value to normalize for differences in electroporation efficiency. Means of a representative experiment are shown.

Several groups have identified CypA as an indispensible cellular factor for efficient viral replication [9]–[11]. Since DEB025 is a cyclophilin-binding molecule, we studied whether DEB025^res^ HCV replicon RNA is still able to replicate in a cell line in which CypA was stably knocked down (KD). The efficiency of the knock down of CypA was verified by means of Western blotting [9]. The replication fitness of WT and DEB025^res^ RNA, transiently transfected into CypA KD and control (CTRL) cells, was evaluated (Figure 5). DEB025^res^ replicon proved less fit than WT replicon in the CTRL cells (on average 6-fold lower than WT-value at 96h post transfection). A replication-deficient replicon (the ‘GND-variant’), was included as an internal control. Conversely DEB025^res^ replicon had markedly increased replication fitness in the CypA KD cells compared to WT (on average 8-fold WT-value at 96h post transfection). This indicates that mutations in the DEB025^res^ replicon render HCV replication less dependent on CypA.

### DEB025 disrupts the interaction between CypA and (WT or DEB025^res^) NS5A

**Figure 6:**
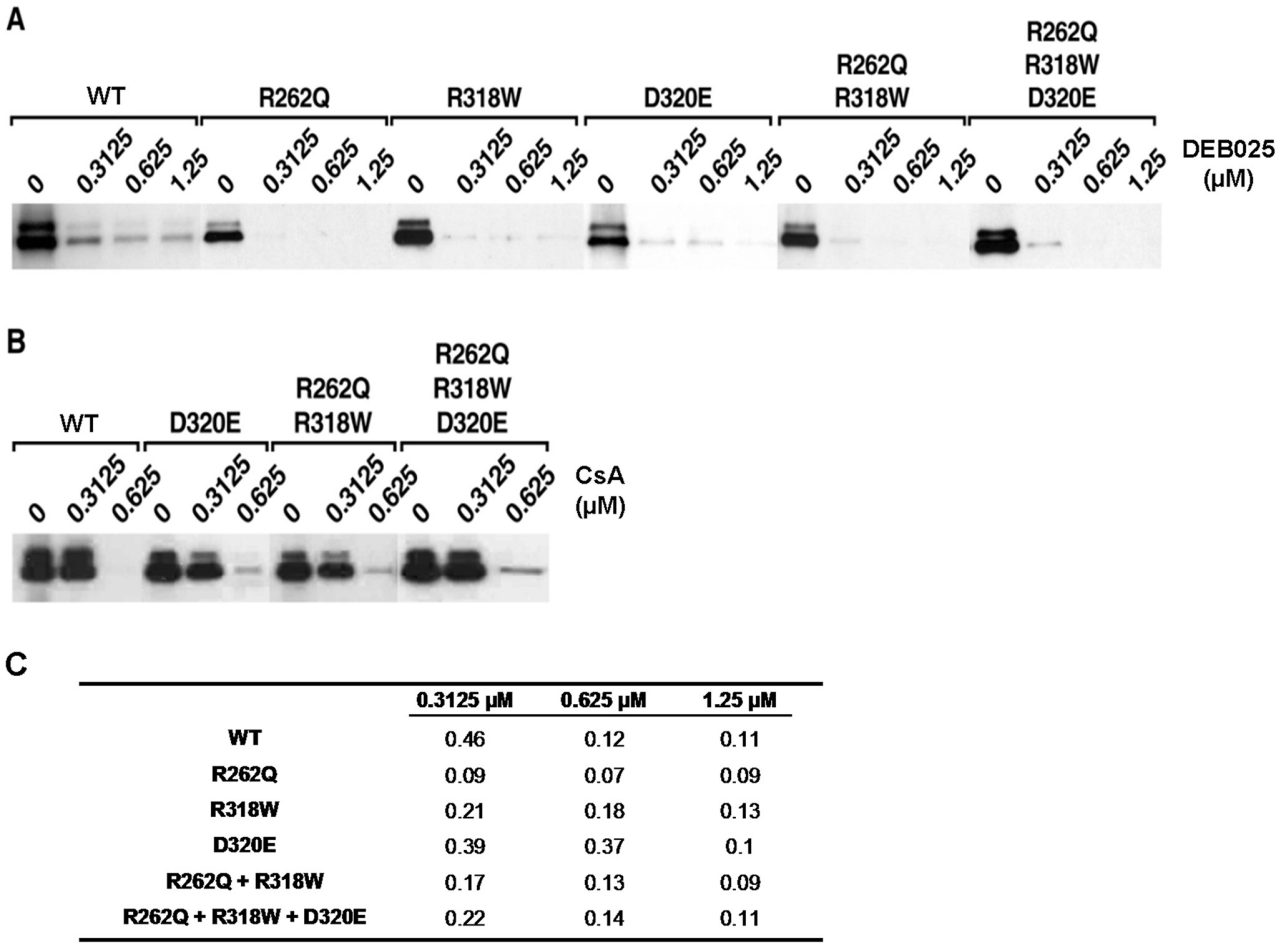
DEB025 disrupts the CypA-NS5A interaction in a dose-dependent manner. Recombinant GST-CypA (100 ng) was incubated with recombinant NS5A Con1-His (10 ng) together with increasing concentrations of DEB025 (A) or CsA (B) for 3 h at 4°C. Glutathione beads were added to the GST-CypA/NS5A-His mixture for 30 min at 4°C and washed. Bound material was eluted and analyzed by Western blotting using anti-His and anti-CypA antibodies. (C) The percentage of NS5A pulled down in the presence of increasing concentrations of DEB025, relative to the amount of NS5A pulled down in the absence of the drug.

Since CypA is crucial for an efficient HCV replication [9], [10], [29] and since direct interactions between domain II of NS5A and CypA have recently been reported [18]; there may be a direct correlation between CypA assistance to HCV replication and CypA binding to NS5A. Based on this model, we postulated that DEB025, as a Cyp inhibitor, would interfere with the interaction between CypA and NS5A, thereby inhibiting HCV replication. We therefore studied whether DEB025 and CsA are able to disrupt the interaction between CypA and NS5A. Pull down experiments between GST-CypA and His-tagged NS5A revealed that DEB025 and CsA inhibit the CypA-NS5A interaction in a dose-dependent manner (Figure 6A). DEB025 was more potent in blocking the CypA-NS5A interaction than CsA (Figure 6B), further supporting the earlier finding that the affinity of DEB025 to CypA is superior to that of CsA to CypA [30]. We confirmed earlier that GST alone does not immunoprecipitate NS5A [16]. We next studied whether the NS5A mutations, either alone or in combination, have an influence on (i) the binding of NS5A to CypA and (ii) the sensitivity of the CypA-NS5A interaction to DEB025. All NS5A mutants studied were found to bind with the same relative interaction to CypA as WT NS5A (Figure 6A). Also the interactions between CypA and WT or mutated NS5A were equally disrupted either by DEB025 or CsA.

**Figure 7:**
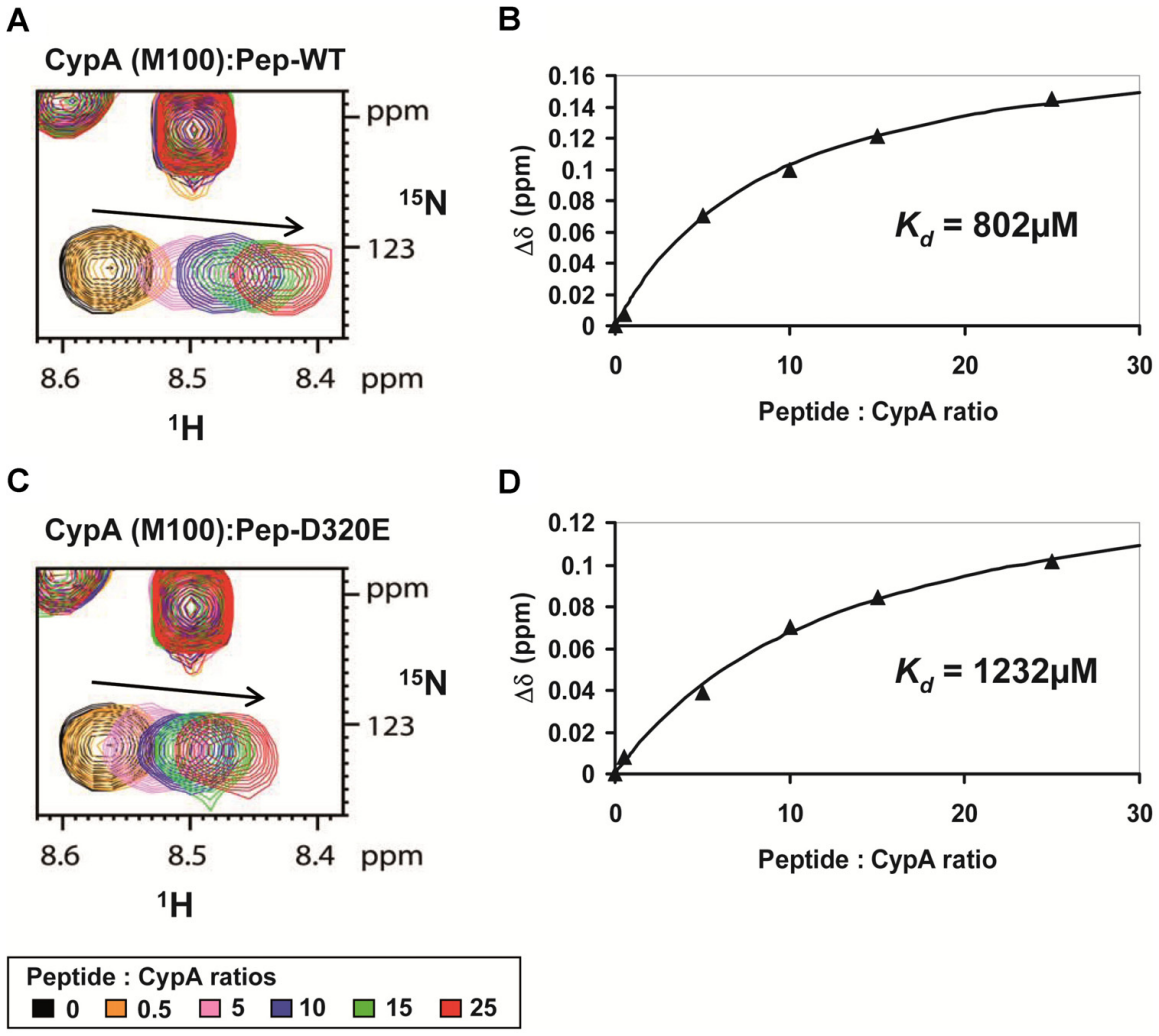
NMR titration experiments between CypA and NS5A-derived peptides, Pep-WT or Pep-D320E. Panels (A) and (C) correspond to the superimposition of the ^1^H,^15^N HSQC spectra of ^15^N-CypA acquired in the presence of increasing amounts of unlabeled peptides; Pep-WT in (A) and Pep-D320E in (C). Whereas both peptides correspond to residues 308–327 of HCV NS5A protein (GT1b, Con1), Pep-WT correspond to the WT sequence (308-KFPRAMPIWARPDYNPPLLE-327) and Pep-D320E carries the D320E mutation (308-KFPRAMPIWARPEYNPPLLE-327). M100 in CypA is a residue that is located at the periphery of the PPIase active site. (B) and (D), titration curves corresponding to experiments in (A) and (C) respectively. The ^1^H,^15^N combined chemical shift perturbations δΔ (in ppm) (δΔ = |δ(^1^HN)| + 0.2x|δ(^15^N)|) were plotted as a function of the Peptide:CypA molar ratios. The dissociation constants (*K_D_*) were obtained by fitting the experimental data with the following equation: *K_D_ = *[CypA_free_]x[Peptide_free_]/[CypA:Peptide].

To further quantify this observation, we prepared by chemical synthesis two peptides (20-mers) with the sequences of the WT domain II of NS5A (Pep-WT) or the DEB025^res^ D320E mutant (Pep-D320E). We centralized the 320 position in these 20-mer peptides, while maintaining the up- and downstream proline residues. It was previously shown that this motif in domain II of NS5A interacts with CypA [18]. NMR spectra of both peptides coincided reasonably well with the spectra of the identical sequences in the full-length domains II, underscoring the natively unfolded character of domain II of NS5A [18]. We then titrated these peptides against a ^15^N-labeled CypA sample, and derived from the chemical shift differences a dissociation constant (Figure 7A–D). Chemical shift perturbations were mainly monitored for residues in the active site of CypA. The dissociation constants derived for both peptides were weak (with K_D_ values of 0.802 mM and 1.232 mM for the Pep-WT and Pep-D320E peptide, respectively), probably reflecting the fact that we did not use the full-length protein as substrate. Importantly, the mutation did not dramatically alter the dissociation constants, in agreement with the results from the pull-down experiments.

### A peptide carrying the D320E mutation has a markedly altered *cis*/*trans* configuration than WT

**Figure 8:**
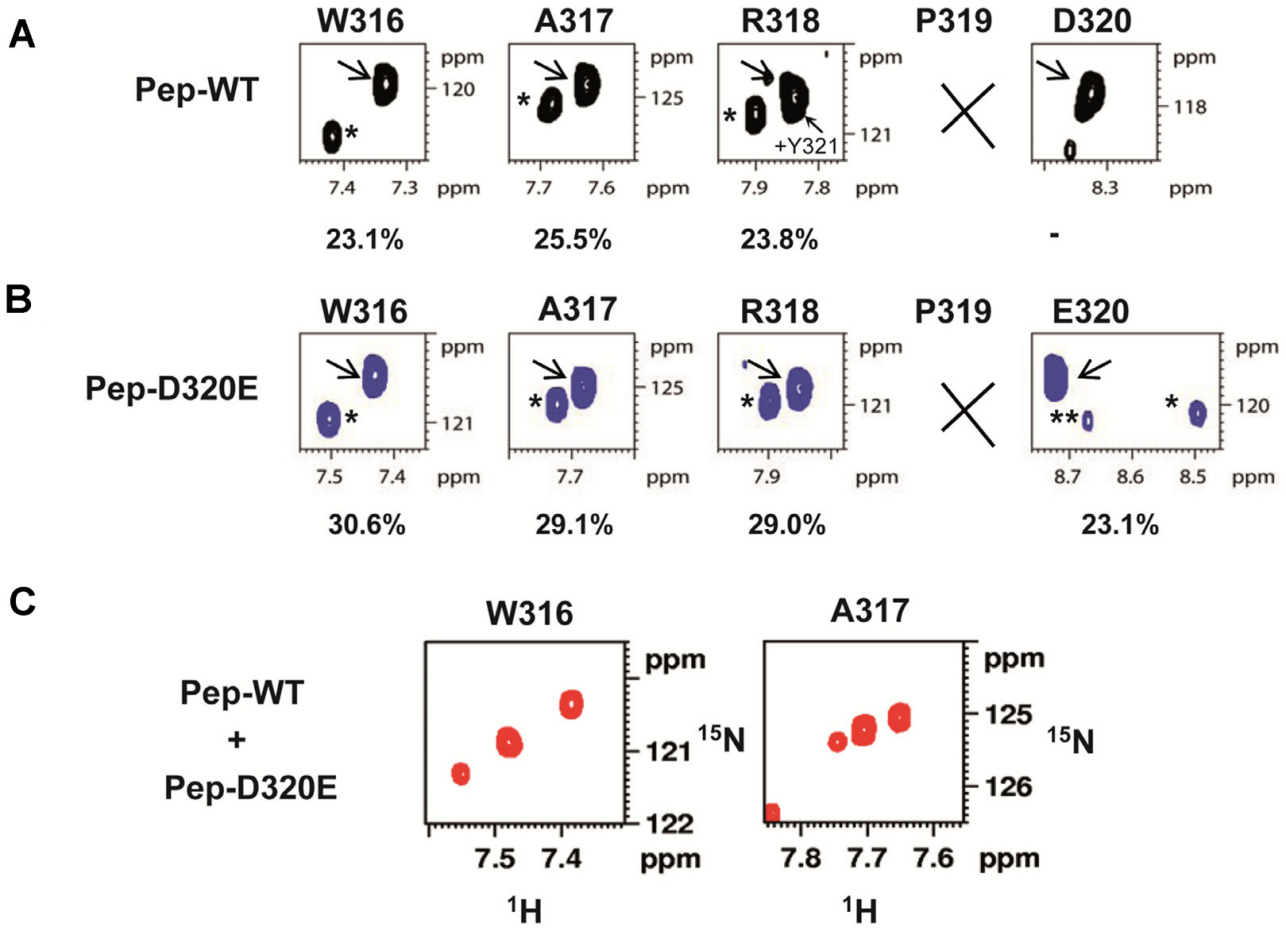
NMR analysis of the conformational consequences of the D320E mutation. ^1^H,^15^N HSQC spectra were acquired at natural abundance on Pep-WT (A) and Pep-D320E (B). The NMR backbone proton amide resonances corresponding to each residue in the 316-WARP(D/E)-320 motif are shown in the different inserts. The resonances indicated by an arrow correspond to the major form of the residue whereas the ones marked with an asterisk correspond to the minor form. The population of the minor form is given as a percentage of the total peptide population. (C) ^1^H,^15^N HSQC spectrum acquired at natural abundance on a mixture of Pep-WT and Pep-D320E peptides at equimolar ratio (3 mM each).

Because the peptides did not show an altered affinity for the CypA surface, we further recorded heteronuclear ^1^H,^15^N HSQC spectra at natural abundance on them. NMR is unique in the sense that it can detect the *trans* and *cis* conformations of any prolyl bond, even though these leave no mass signature. In the WT peptide, we observed a major and minor conformation for several residues preceding the Pro319 (Figure 8A and B), that we tentatively assigned to the conformers associated with the peptide bond preceding Pro319 in the *trans* and *cis* conformation. Upon integration of these peaks, we obtained equilibrium values of 75.9% for the major and 24.1% for the minor form. When we repeated the same experiment with the D320E peptide, however, we found that the peak resonating at the minor frequency in the WT peptide had become the major one, with populations of 70.4%. To be sure that peak positions had not shifted due to slightly different experimental conditions, we mixed both peptides in equimolar amounts and ran again the NMR spectrum. We now only observed three peaks, showing unambiguously that the minor correlation peak in the WT peptide coincides with the major one in the D320E peptide (Figure 8C). Although further studies on the full-length domain II of the WT and D320E NS5A proteins will be required to elucidate the precise structural origin of this conformational inversion, our observations suggest that CypA resistance arises by mutations that affect directly the conformation of domain II of the NS5A protein. The prolyl *cis-trans* isomerisation activity of CypA would thereby be not strictly required anymore, explaining the resistance to DEB025 or CsA.

## Discussion

We previously reported on the potent anti-HCV activity of the non-immunosuppressive CsA analogue DEB025 [22] and studied the specific *in vitro* characteristics of this molecule when combined with pegIFN/RBV and several DAA inhibitors [23]. Here we report the *in vitro* characterization of DEB025^res^ replicon and propose a mechanism by which DEB025, and by extension other cyclophilin-binding agents with anti-HCV activity, inhibit viral replication.

The selection of resistance to either DEB025 or CsA proved to be a lengthy process, indicating that the genetic barrier towards resistance of this class of inhibitors is high. This may be explained by the unique mechanism of action of these inhibitors. Indeed, the virus does not need to acquire (a) mutation(s) that prevent(s) direct binding of the inhibitor to the viral protein, but must rather acquire a mutation that makes it less dependent of a host factor that is otherwise essential for viral replication. We reported that DEB025 is highly efficient in curing hepatoma cells from their replicon [23]. Both in terms of drug concentration and time of culture needed to result in clearance, DEB025 is by far more efficient than ‘classical’ DAA inhibitors. This may be related to the fact that drug-resistant variants are not readily selected. Moreover, this might also be explained by the fact that DEB025 specifically sequesters CypA and therefore prevents conformational changes in the replication complex that are crucial for viral replication. Altering the conformation of replication complex components may result in an irreversible modification (which is not the case with DAA inhibitors) and thus efficient clearance. Earlier clinical studies have shown that resistance mutations to protease inhibitors emerged in patients within days of treatment [31], leading to treatment failure. The low barrier to resistance to protease inhibitors and other DAAs therefore requires combination therapy. Since DEB025 is fully active against resistant replicons selected by other DAAs [23] and DAA inhibitors retained WT-activity against these DEB025^res^ or CsA^res^ replicons, the high resistance barrier and favorable safety profile of DEB025 potentially allow it to be the backbone of future combination therapy as well as an essential component of rescuing regimen for patients who failed protease inhibitors or other DAAs treatment.

In this study, transfection of RNA isolated from these DEB025^res^ or CsA^res^ cultures into naïve hepatoma cells transferred part of the originally observed resistance, indicating that resistance can be conferred by the viral genome. Four independent resistance selection assays were carried out; with NS5A D320E being the only mutation identified in all resistant replicons. A number of other NS5A mutations at relatively conserved sites were also seen in one of the four resistant lines; these are not known as adaptive mutations that generally enhance replication. However, reintroduction of single as well as combinations of mutations into a WT background identified D320E as the single mutation conferring part of the observed genome-associated resistance. The region around the D320 position is well-conserved among a diverse set of HCV genotypes. This suggests that it might play an important role in some aspect of the HCV life cycle [32]. In addition, other groups recently reported that D320E and the near variant Y321N are involved in resistance to cyclosporins *in vitro* [13], [14], [17].

However the full extent of genome-associated resistance was not restored by the single mutation D320E. Exchanging the NS5A gene from WT replicons by the corresponding NS5A from DEB025^res^ replicon resulted in a yet more pronounced resistance. A number of other mutations could be found in subgenomic replicons (relative to the original Con1 sequence), which generally enhance subgenomic replication. Although these mutations alone were unable to confer resistance to DEB025 or CsA, they did contribute, in concert, to the elevated resistance level as was evident in the swapping experiments. It was demonstrated that CypA binds to NS5B and that the enzymatic pocket of CypA is critical for this interaction. DEB025 was reported to prevent the CypA-NS5B interaction [9]. Moreover, it has been suggested that NS5B is involved in the mechanism of action of anti-HCV activity of Cyp inhibitors [28]. However our observations argue against this hypothesis (at least in the context of GT1b), as introducing the NS5B gene derived from DEB025^res^ replicon into a WT background did not result in transfer of resistance. Also I432V in NS5B, a variant previously reported as being involved in CsA resistance [28], was not able to confer CsA or DEB025 resistance in our hands.

The HCV NS5A protein has no known intrinsic enzymatic activity, but likely exerts its functions through interactions with viral and cellular factors. It is a pleiotropic protein which plays an essential role in the HCV viral life cycle for example by supporting viral RNA replication as well as by modulating the physiology of the host cell to favour viral replication [33]. It occurs in a basally- and hyperphosphorylated form, with different putative functions during HCV replication [34]. NS5A consists of three domains, separated by low-complexity sequences [35]. Whereas domain I appears to be involved in RNA-binding [36]–[38] and domain III is essential for infectious particle assembly [39]–[41], the specific role of domain II, which is natively unfolded [42], [43], still remains to be elucidated. Direct molecular interactions between domain II and both CypA and CypB have been observed [18]. Surprisingly, we observed that the interaction of CypA with DEB025^res^ NS5A was as sensitive to inhibition by DEB025 and CsA as the interaction between CypA and WT NS5A. This result implies that DEB025^res^ NS5A does not depend on CypA-binding to efficiently contribute to RNA replication. Small molecule inhibitors of HCV that target NS5A have been shown to reduce the hyperphosphorylated form of NS5A [44]. Since DEB025^res^ replicons carry mutations in NS5A, we studied by means of Western blot and phosphatase treatment experiments whether NS5A hyperphosphorylation was different in either WT, CsA or DEB025^res^ cultures. No such differences were however observed (data not shown). Mutations conferring resistance to these NS5A inhibitors reside in domain I of NS5A, so cross-resistance between DEB025 and this type of inhibitors is unlikely to occur [45].

Replication of the DEB025^res^ genome proved, unlike replication of the WT genome, to be efficient in CypA KD cells, indicating that the drug resistant genome is less dependent on CypA (and thus likely on the *cis-trans* isomerase activity) for efficient replication. Given the fact that domain II of NS5A contains many proline residues that form potential valid substrates for the enzymatic peptidyl-prolyl *cis-trans* isomerase activity of cyclophilins, we studied by means of NMR the conformation of a 20-mer peptide carrying the E320 in the middle and compared this to that of a corresponding WT peptide. The residues preceding Pro319 in the WT peptide occurred on average for 24.1% in a minor conformation that we tentatively assigned to the Pro319 *cis* form. Based on the chemical shift identity, this same minor conformer in the WT-peptide becomes the dominant one in the D320E-peptide, with a relative population of 70.4%. We therefore assume that the DEB025^res^ genome lowers CypA dependence by acquiring a conformational inversion mediated by the D320E mutation, that otherwise is supported by CypA. The data generated with these peptides may not necessarily reflect the situation of the full length NS5A protein in the host cell. However, based on the dataset obtained, it can be hypothesized that the change in conformation is in some way crucial for HCV replication. Blocking the interaction between CypA and NS5A by a cyclophilin-binding compound such as DEB025, results therefore in inhibition of HCV replication. It has been reported that NS5A interacts with NS5B through two independent regions (of which one includes the D320 position) and that NS5A modulates the activity of NS5B RdRp through this interaction [46]. Possibly the conformational changes in NS5A catalyzed by CypA contribute to these crucial interactions with NS5B.

Taken together, our data point to an entirely novel and exciting mechanism by which HCV (GT1b) replication can be blocked by CypA inhibitors. Resistance mutations may trigger a particular conformation in NS5A that is (directly or indirectly) required for efficient viral replication. The fact that the virus needs to develop a strategy to efficiently replicate largely independent from a host factor may explain the high genetic barrier that has to be passed to develop resistance against cyclophilin inhibitors.

The distinctive resistance profile of DEB025 provides a unique option in treating chronic HCV infection, both as the backbone of future combination therapy with other compounds in treatment-naïve patients and as rescue therapy for patients harbouring resistance mutations to other classes of anti-HCV agents.

## Materials and Methods

### Compounds

The compounds used were described previously [23].

### Cell lines

Huh7 cells containing subgenomic HCV genotype 1b replicon I_377_/NS3-3′/wt (Huh 9–13) [47], [48] and I_389_luc-ubi-neo/NS3-3′/5.1 (Huh 5–2) were used to select for resistance. Transfection experiments were performed in Huh7-Lunet cells [49] supporting high level of viral RNA replication. The generation of CypA knock down (KD) cells has been described before [9]. Stable Cyp KD cell lines were obtained under puromycin (1 µg/ml) selection and CypA KD was verified by Western blotting as described previously [9]. Cells were grown in Dulbecco's modified Eagle's Medium (DMEM; Gibco) supplemented with 10% heat-inactivated fetal bovine serum (FCS) (Integro), 1x non-essential amino acids (Gibco), 100 IU/ml penicillin (Gibco), 100 µg/ml streptomycin (Gibco), 250 µg/ml G418 for Huh 5-2 and 1000 µg/ml G418 for Huh 9–13 cells or puromycin (1 µg/ml) for Cyp KD cells.

### Selection of drug resistant replicon cell lines

Drug resistant replicons were generated by passaging HCV subgenomic replicon containing cells (Huh 9–13 cells) under G418 selection (1 mg/ml G418) in the presence of gradually increasing concentrations of DEB025 or CsA. When cells suffered from compound pressure, G418 pressure was removed till cells recovered. Thereafter, cells were re-cultured with either CsA or DEB025 and G418 pressure. Four independent resistance selection experiments were carried out, three with Huh 9–13 replicons and one with Huh 5–2 replicons.

### Antiviral assays and RT-qPCR

HCV replication assays were performed and analysed using published procedures [23].

### Replicon sequencing

Total RNA from pooled drug resistant or control (non-treated) cultures was isolated (RNeasy mini kit, Qiagen), according to the manufacturer's instructions, and amplified by RT-PCRs to generate overlapping fragments that covered the full length of the replicon. The sequence of the entire region encoding HCV non-structural proteins was determined. Alternatively, the RT-PCR fragments from the NS5A region were cloned into a pCRII-TOPO TA vector (Invitrogen) and plasmid DNA from 10 bacterial colonies was sequenced (Big Dye® Terminator v3.1, Applied Biosystems). The sequences were aligned and analysed using Vector NTI advance software (Invitrogen).

### Site-directed mutagenesis

Plasmid pFKI_389_ Lucibineo EI NS3-3′ET [50] was used for the reintroduction of identified mutations. Initially, a NotI and MluI fragment containing the NS3, NS4A, NS4B and part of the NS5A coding region was subcloned into the pCRII-TOPO vector (Invitrogen) for reintroduction of the A241P mutation in NS3. A MluI and XhoI fragment that contains a part of the NS5A coding region was subcloned into a pCRII-TOPO vector in which a part of the yellow fever virus was inserted to provide the vector with the correct restriction sites. This vector was used to reintroduce the R262Q mutation in NS5A. An XhoI and SpeI fragment containing part of the NS5A and the NS5B coding region was subcloned into the pCRII-TOPO vector for reintroduction of the R318W and D320E mutations in NS5A. Site-directed mutagenesis was performed using the “QuickChange Site Directed Mutagenesis Kit” (Stratagene) according to manufacturer's instructions. The primers used for mutagenesis are shown in Table S1. The mutated fragments were cloned back into the pFKI_389_ Lucubineo EI NS3-3′ET plasmid. DNA sequencing confirmed the presence of the introduced mutations.

### NS5A/NS5B swapping

RNA was isolated from either DEB025^res^ or WT HCV subgenomic replicon cells using the RNeasy mini kit (Qiagen). First-strand cDNA was synthesized using SuperScript II RT (Invitrogen). The NS5A or NS5B-containing regions were amplified in an AccuPrime Pfx DNA polymerase reaction (Invitrogen) for subsequent cloning into the WT Con1 plasmid. All constructs were sequenced to verify the correct manipulation.

### *In vitro* transcription

Five µg of mutated or WT plasmids were linearized with ScaI (Promega) and AseI (New England Biolabs) (only for pFKI_389_ Lucibineo EI NS3-3′ET plasmid). Thereafter, linearized plasmid was phenol/chloroform extracted, ethanol precipitated and dissolved in RNase free water. HCV replicon RNAs were generated using the RiboMAX™ Large scale RNA production system-T7 (Promega). RNA was purified and collected by using the RNeasy mini kit (Qiagen). The concentration and purity of RNA were spectrophotometrically measured.

### Transfection

Replicon RNA was delivered to Huh7-Lunet cells by electroporation or liposome-mediated transfection (for the generation of stable cell lines).

#### Lipofection

One day before lipofection, Huh7-Lunet cured cells were seeded in a 25 cm^2^ flasks at a cell density of 5.26×10^5^ cells. Just before transfection, the cells were washed once with opti-MEM® I (Invitrogen) and incubated for 4 h at 37°C with transfection medium containing: 26.3 µg of RNA, DMRIE-C Reagent (Invitrogen) and opti-MEM I serum free medium. Four hours post transfection, medium was replaced by culture medium without G418. Depending on the proliferation of the cells, G418 was gradually added to the cultures (to a final concentration of 1 mg/ml) to generate stable cell lines.

#### Electroporation

Huh7-Lunet single-cell suspensions were prepared by trypsinization, washed twice with Opti-MEM® I (Invitrogen) and then resuspended at 1×10∧7 cells per ml in Cytomix [51] containing 2 mM ATP and 5 mM glutathione. Ten µg of *in vitro* transcribed RNA was mixed with 400 µl cell suspension in a cuvette with a gap width of 4 mm (VWR International) and an ECM 830 Electro Square Porator™ (BTX Harvard Apparatus) was used to deliver 5 pulses at 900 V, 99 µsec. Cells were immediately transferred to 20 ml complete DMEM and seeded as required for the assay. Briefly, 100 or 700 µl aliquots of the cell suspension were seeded in a 96- or 24-well plate (Iwaki), respectively, filled with serial dilutions of the antiviral molecule in complete DMEM. Cells were allowed to proliferate for 4 days at 37°C, after which the luciferase signal was determined using the Steady-Glo luciferase assay system (Promega) in a Luminoskan Ascent (Thermo).

### Replication fitness

Transfections were performed in Huh7-Lunet cells as described above with the exception that cells were transfected with 5 µg RNA and 5 µg tRNA as a carrier (Sigma Aldrich). Tranfected cells were immediately transferred to 24 ml of complete DMEM and a 1.5 ml aliquot of the cell suspension was added to a 6 well plate (Iwaki). Cells were collected at 4 h (normalization point) and 1, 2, 3, 4 days post transfection to compare mutant luciferase values with WT values. Cells only electroporated with tRNA were analyzed at the indicated time points for luciferase activity and used as background activity from the residual input RNA. A GND replicon (a replication-deficient subgenomic replicon encoding a GDD to GND mutation in NS5B [52]) served as a negative control.

### Production of recombinant CypA and NS5A proteins

Recombinant GST (glutathione S transferase)-CypA was produced and purified as we described previously [53], as well as full-length NS5A GT1b (pET-Ub-NS5A Con1-His) was expressed as described previously [54]. NS5A mutants were created by site-directed mutagenesis.

### CypA-NS5A pull-down studies

Pull-down experiments were conducted as described previously [55]. Briefly, GST-CypA (100 ng) was mixed with 10 ng of NS5A-His in a total volume of 200 µl for 3 h at 4°C on a rotating wheel. Glutathione beads (25 µl) were added to the GST-CypA/NS5A-His mixture for 30 min at 4°C and samples were washed 3 times thereafter. Beads were pelleted for 30 sec at 2000 g in a microcentrifuge and bound material was eluted with 25 µl of 2x SDS sample buffer, heated for 5 min, and frozen at −20°C. Bound material was then analyzed by Western blotting using anti-CypA and anti-His antibodies as described previously [9]. CsA or DEB025 was added together with NS5A and CypA. To calculate the percent of NS5A pulled down relative to the amount of NS5A pulled down in the absence of the drug scans of ECL-developed films were analyzed by Bio-Rad densitometer model 620 and quantified by Bio-Rad Program Manager software (Bio-Rad Laboratories, Inc., Hercules, CA). Data are expressed in percentage of pulled down NS5A in the presence of increasing concentrations of DEB025 by arbitrarily fixing the percentage (band intensity) of each pulled down NS5A protein in the absence of the drug at 100.

### NS5A-derived peptides NMR analysis

Purified synthetic peptides, Pep-WT (308-KFPRAMPIWARPDYNPPLLE-327 and Pep-D320E (308-KFPRAMPIWARPEYNPPLLE-327), corresponding to residues 308–327 of NS5A from HCV GT1b (Con1) were obtained from Neosystems (Strasbourg, France). The purity of the peptide was verified by high pressure liquid chromatography and mass spectrometry as greater than 95%. Recombinant ^15^N-labelled CypA was produced and purified as described previously [18]. Spectra were acquired on a Bruker Avance 600 MHz equipped with a cryogenic triple resonance probe head (Bruker, Karlsruhe, Germany). The proton chemical shifts were referenced using the methyl signal of TMSP (sodium 3-trimethylsill-[2,2_,3,3_-d4]propionate) at 0 ppm. Spectra were processed and analyzed with the Bruker TopSpin software package 2.1. To study the interaction between CypA and peptide Pep-WT or Pep-D320E, ^15^N-labelled CypA (100 µM) was mixed with increasing concentrations of non labelled peptide (from 0 to 2.5 mM) and a ^1^H,^15^N HSQC spectrum was recorded at each step. The combined chemical shift perturbations (δΔ, in ppm) following peptide addition were calculated as δΔ = |δ(^1^HN)| + 0.2x|δ(^15^N)| whereby δ(^1^HN) and δ(^15^N) are the chemical shift perturbations in the ^1^H and ^15^N dimensions, respectively. To study the solution behaviour of Pep-WT and Pep-D320E, each peptide (10mM) was analyzed by NMR at natural isotopic abundance. Proton amide resonances were assigned by classical procedure using homonuclear ^1^H,^1^H TOCSY and ^1^H,^1^H NOESY experiments. To obtain the population of the major and minor form of the peptides we measured the integrals of the proton amide resonances on a ^1^H, ^15^N HSQC spectrum. In order to exclude sample variations as a reason for the small shifts between resonances, a mixture of both peptides (3mM each) was analyzed in a similar manner.

## Supporting Information

Table S1 Primers used for site-directed mutagenesis.
(2.72 MB TIF)

Table S2 Prevalence of the variants found in the replicon after DEB025 selection in different genotypes in the Los Alamos database.
(1.80 MB TIF)

Table S3 Mutations identified in independently selected DEB025 or CsA resistant replicons.
(1.53 MB TIF)

Figure S1 Dose-response curves for inhibition of replicon replication by DEB025 (A) or CsA (B) in Huh7-Lunet cells transiently transfected with mutant replicon RNA (indicated on the right site of each panel). HCV replicon RNA was quantified by means of a luciferase assay and data are expressed as percentage of untreated controls. Data are mean values ± standard deviations for at least two independent experiments.
(0.94 MB TIF)

Figure S2 Dose-response curves for inhibition of replicon replication by DEB025 in Huh7-Lunet cells transiently transfected with mutant replicon RNA (indicated on the right site of each panel). HCV replicon RNA was quantified by means of a luciferase assay and data are expressed as percentage of untreated controls. Data are mean values ± standard deviations for at least two independent experiments.
(0.59 MB TIF)
