# Supplementary figures and images for: DEB025 (Alisporivir) Inhibits Hepatitis C Virus Replication by Preventing a Cyclophilin A Induced Cis-Trans Isomerisation in Domain II of NS5A
Source: PLoS One. 2010 Oct 27;5(10):e13687. doi: 10.1371/journal.pone.0013687 (PMC2965138; doi:10.1371/journal.pone.0013687)

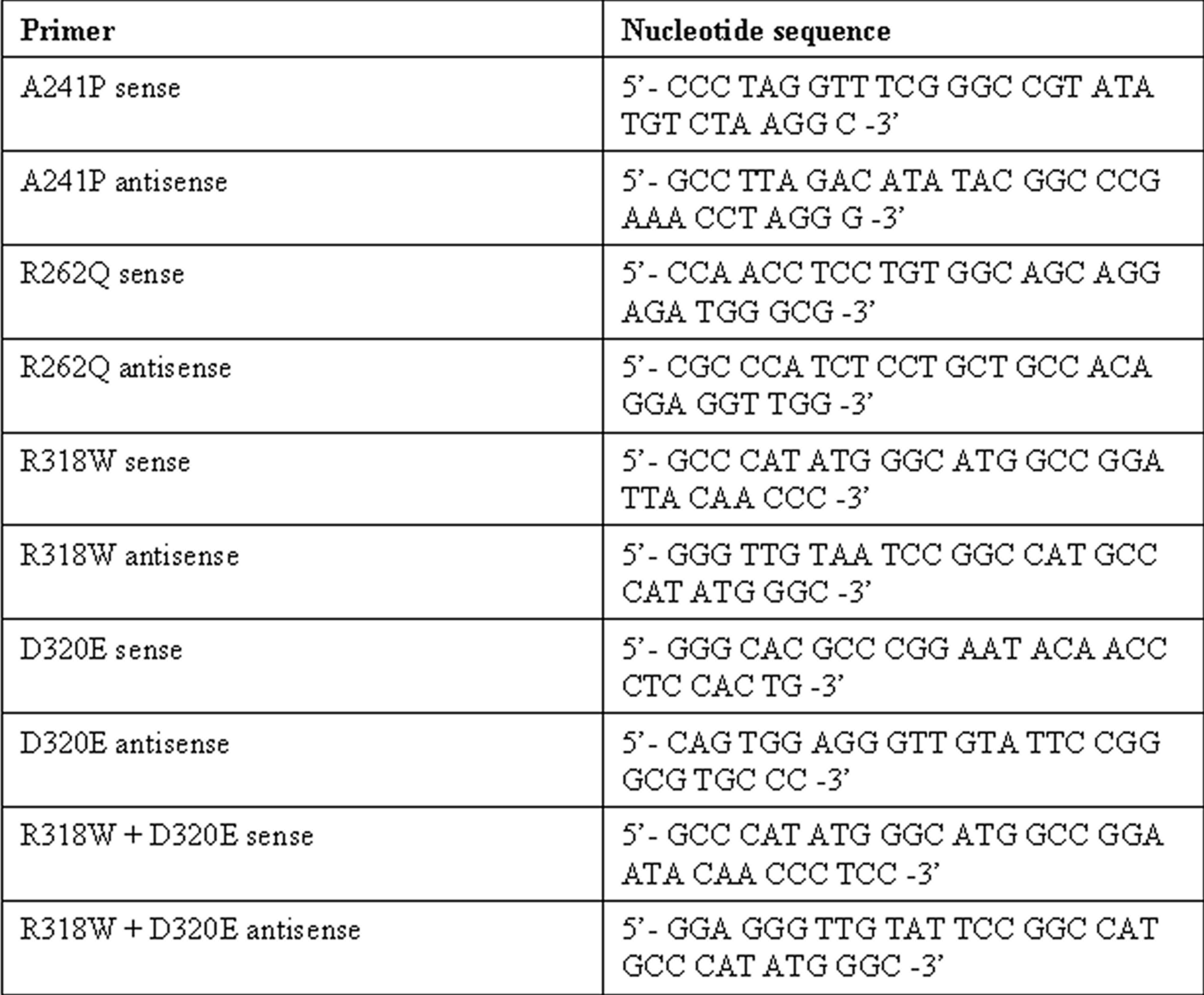

Supplement: Table S1 — Primers used for site-directed mutagenesis. (2.72 MB TIF) [file pone.0013687.s001.tif]

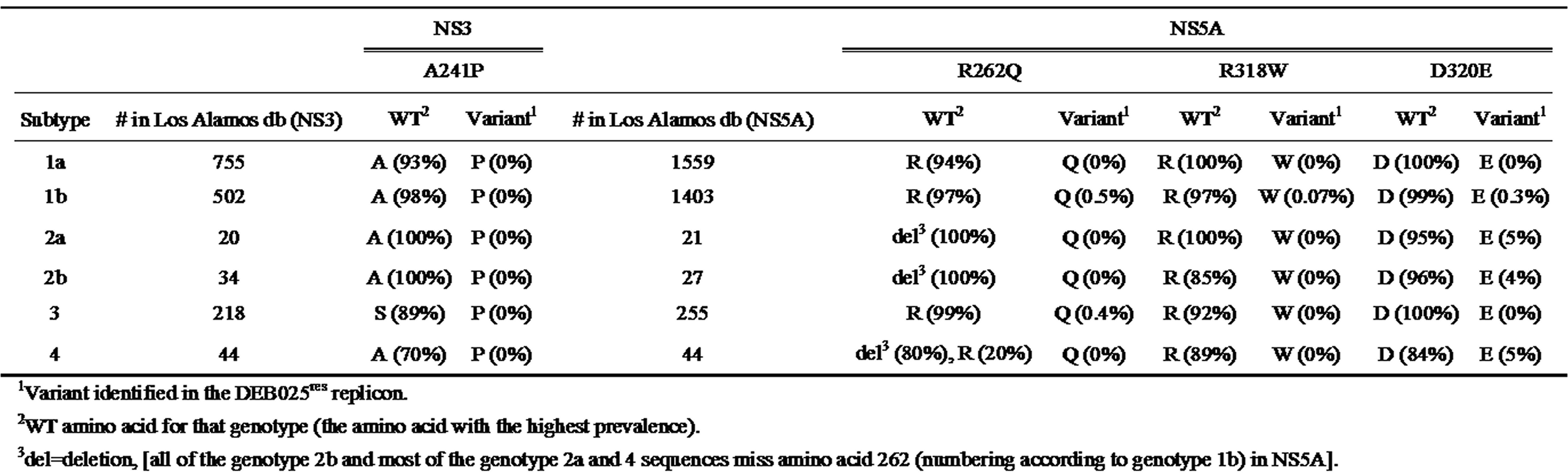

Supplement: Table S2 — Prevalence of the variants found in the replicon after DEB025 selection in different genotypes in the Los Alamos database. (1.80 MB TIF) [file pone.0013687.s002.tif]

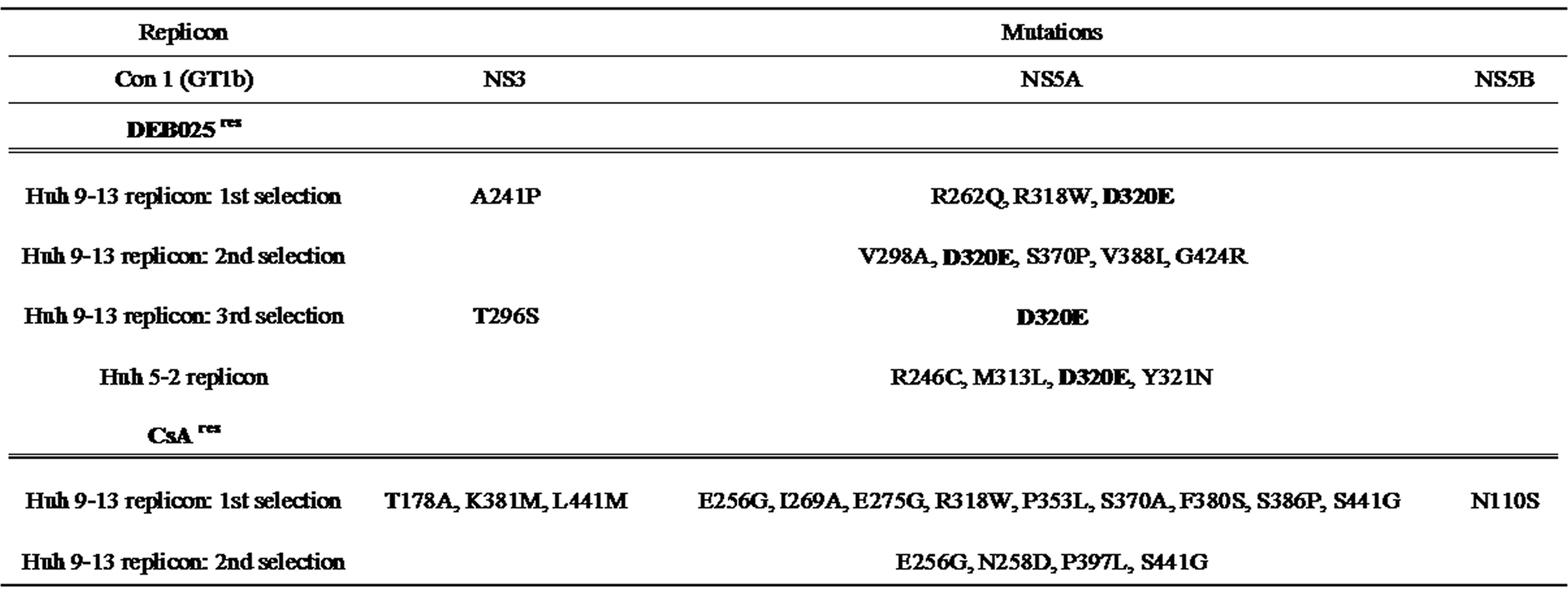

Supplement: Table S3 — Mutations identified in independently selected DEB025 or CsA resistant replicons. (1.53 MB TIF) [file pone.0013687.s003.tif]

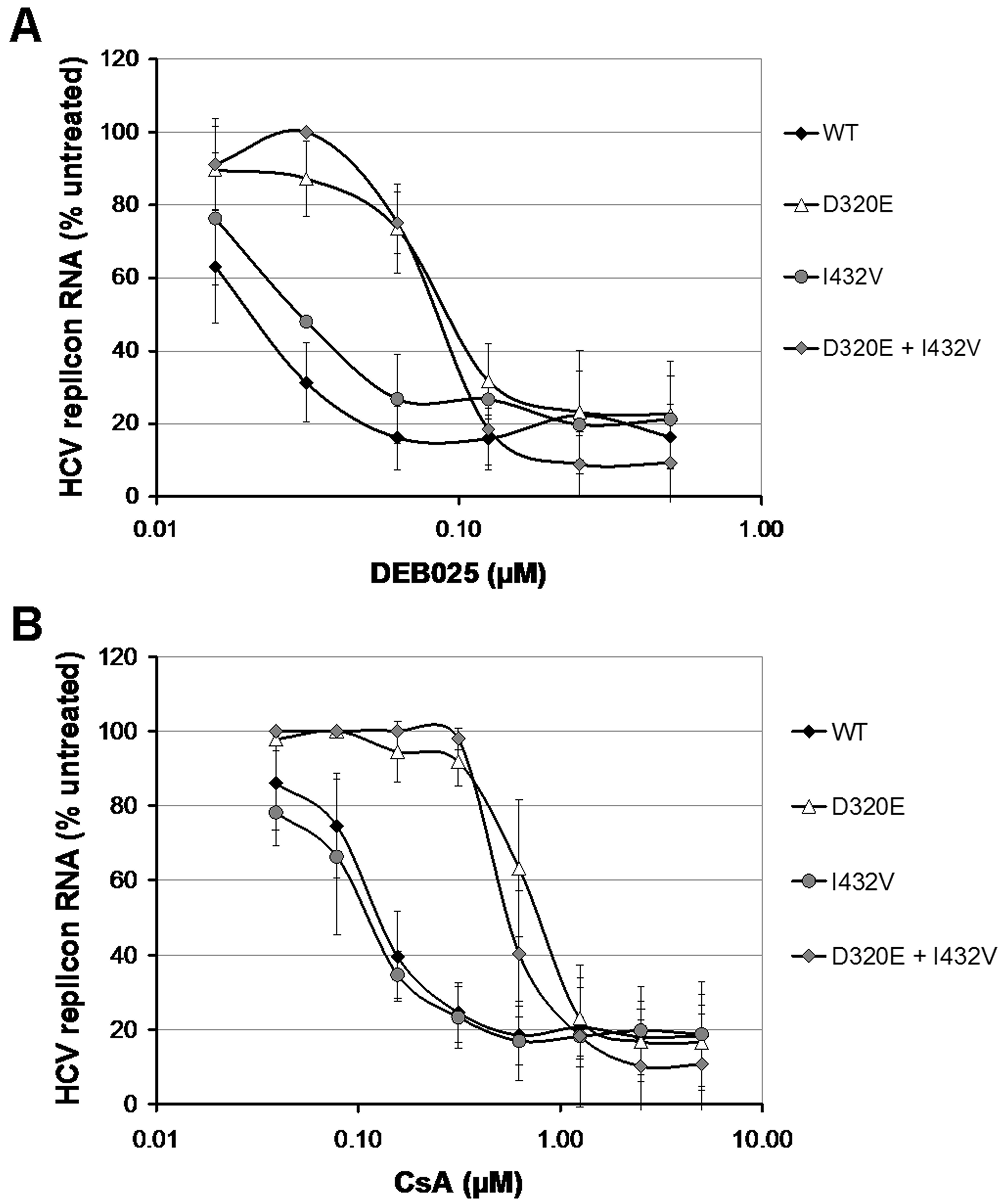

Supplement: Figure S1 — Dose-response curves for inhibition of replicon replication by DEB025 (A) or CsA (B) in Huh7-Lunet cells transiently transfected with mutant replicon RNA (indicated on the right site of each panel). HCV replicon RNA was quantified by means of a luciferase assay and data are expressed as percentage of untreated controls. Data are mean values ± standard deviations for at least two independent experiments. (0.94 MB TIF) [file pone.0013687.s004.tif]

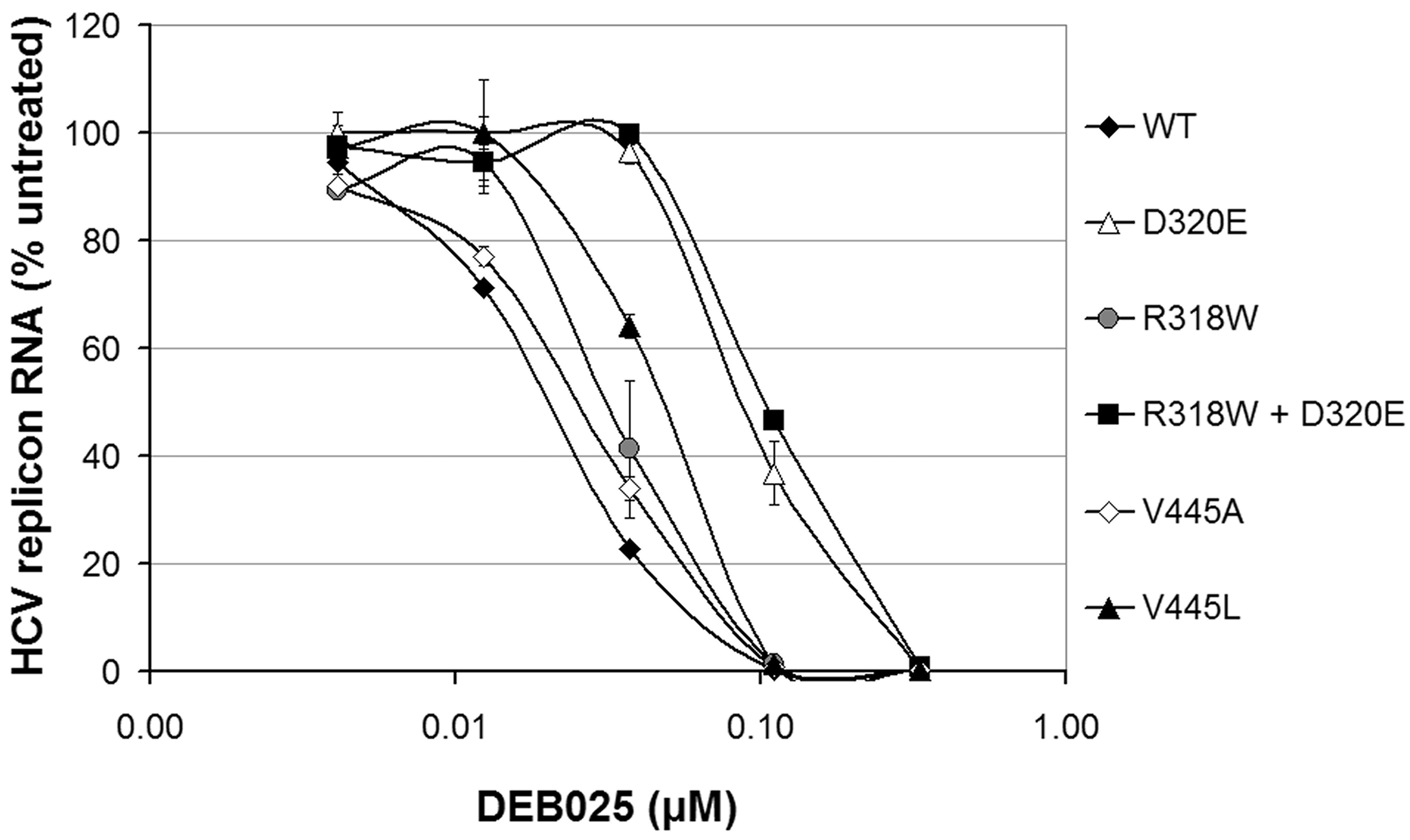

Supplement: Figure S2 — Dose-response curves for inhibition of replicon replication by DEB025 in Huh7-Lunet cells transiently transfected with mutant replicon RNA (indicated on the right site of each panel). HCV replicon RNA was quantified by means of a luciferase assay and data are expressed as percentage of untreated controls. Data are mean values ± standard deviations for at least two independent experiments. (0.59 MB TIF) [file pone.0013687.s005.tif]
